# Exposure to the new Medicare Advantage risk adjustment model varies across insurers

**DOI:** 10.1093/haschl/qxag092

**Published:** 2026-04-16

**Authors:** Jeffrey Marr, Andrew M Ryan, David J Meyers

**Affiliations:** Department of Health Services, Policy and Practice, Brown University School of Public Health, Providence, RI 02903, USA; Department of Health Services, Policy and Practice, Brown University School of Public Health, Providence, RI 02903, USA; Department of Health Services, Policy and Practice, Brown University School of Public Health, Providence, RI 02903, USA

**Keywords:** Medicare, Medicare Advantage, risk adjustment

## Abstract

**Introduction:**

Medicare Advantage plan payment depends on the health of enrolled patients. As a result, the extent to which beneficiary clinical severity is documented administratively—known as coding intensity—is greater in Medicare Advantage (MA) than in traditional Medicare, which inflates payment to plans. In 2024, the Centers for Medicare and Medicaid Services began phasing in a new risk adjustment model intended to reduce the susceptibility of MA payments to higher coding intensity.

**Methods:**

Using 2021 data, we compared average MA contract risk scores under the new model and the old model.

**Results:**

Risk scores were 5.8% lower under the new risk adjustment model. Differences between average risks scores under the new and old model varied substantially across contracts and insurers. For example, 1 large insurer's risk scores were essentially unchanged across models while another large insurer's risk score was 18% lower under the new model. Contracts with higher estimated coding intensity had greater exposure to the new risk adjustment model.

**Conclusion:**

Our results suggest that the new risk adjustment model will likely reduce MA payments due to enhanced coding intensity, with these reductions appropriately targeting insurers that code more intensely.

## Introduction

In Medicare Advantage (MA), which now enrolls over half of Medicare beneficiaries, plan payments are adjusted based on the health of beneficiaries.^1,2^ Plans receive higher payments for patients with greater documented clinical severity who are expected to cost the plan more to cover. Per member per month payments are modified by each beneficiaries' risk score, which is determined by plan submitted diagnosis codes that are aggregated into a composite risk score known as the hierarchical condition category (HCC) score.^2^ This score is based on a model that is trained on traditional Medicare beneficiaries and is designed to predict traditional Medicare spending for patients who have different diagnosed health conditions.^3^ The core assumption of using this risk score to modify plan payments in MA is that a beneficiary in MA with a given set of diagnosed conditions has the same underlying health—and therefore expected spending—as a comparable beneficiary in traditional Medicare.^4^

However, plans have a strong incentive to maximize the number of payment-related diagnoses that are submitted to the Centers for Medicare and Medicaid Services (CMS). There is strong empirical evidence that MA beneficiaries have diagnoses coded with greater intensity than traditional Medicare beneficiaries.^4-12^ There is also evidence of substantial variation across insurers within the MA program, with some insurers exhibiting particularly high coding intensity.^2,13^ Plans may achieve this greater coding intensity through a number of mechanisms, including by encouraging health risk assessments in beneficiaries' homes, by conducting chart reviews, and by focusing on the coding of diagnoses where discretion is involved in the decision.^4,7,9,11,14,15^ Regardless of the mechanism, greater coding intensity in MA breaks the fundamental assumption of the risk adjustment model that a beneficiary with a given set of coded diagnoses in MA or TM has the same underlying level of expected health care spending.

In 2025, Medicare Payment Advisory Commission (MedPAC) estimated that higher coding intensity leads MA plans to be paid 10% more for a given beneficiary than would be spent if that same beneficiary enrolled in traditional Medicare. In 2025, this represented $40 billion in federal spending.^3^

In 2024, CMS began phasing in a new risk adjustment model, V28, that is expected to reduce the impact of higher coding intensity on MA spending.^2,15,16^ This new model makes several notable changes to address CMS' long-standing risk adjustment principle that the model should not use diagnoses where there is significant discretion in recording the diagnoses. First, CMS removed several conditions where there was particularly high coding intensity in MA relative to traditional Medicare, including protein calorie malnutrition and angina pectoris. Second, CMS constrained the values of some HCCs for diseases that can be coded with or without complications (diabetes, congestive heart failure), which were susceptible to differential coding practices.^17^

In addition, CMS made other changes that are not directly related to discretionary codes susceptible to higher coding intensity. This included updating the traditional Medicare data used in training the model from 2014-2015 to 2018-2019 and switching from using ICD-9 to ICD-10 codes in training the model. Overall, these changes represent a significant change to how risk is measured in MA.^3,17^ The new model was phased-in gradually with a blend of the new and the old models used during 2024 and 2025 and full implementation of the new model in 2026.^2^

Our objective was to understand how the introduction of the new risk adjustment model is likely to change the allocation of payments in the MA program. Using 2021 data, we examined differences in the risk scores from the new model (V28) and the old model (V24) across MA contracts. We examined differences across the largest insurers and examined the relationship between the change in risk scores with a measure of coding intensity.

## Methods

### Data sources

Our primary data source is Medicode, a research tool created and maintained by Brown University's Center for Advancing Health Policy through Research. This data contains contract-level information on risk coding in MA. This data is based on a 20% sample of 2021 MA encounter data. Diagnoses recorded during this year would have been used for payment in 2022. It includes the average V24 and V28 risk scores for the contract based on the 2021 data. It also includes a measure of coding intensity (see below) and contract level enrollment. Full documentation of this data is available online at the Medicode website.^18^

We supplemented Medicode data with public data on contract characteristics, as well as data aggregated from the 100% Medicare Master Beneficiary Summary file on the contract's demographics.

### Measures

We used 3 main sets of measures in this paper. First, we used the V28 and V24 risk scores. These are contract-level averages of the raw scores calculated from the encounter data rather than the risk scores that are used for payment. However, a critical transformation of these data is needed to represent the risk scores used for payment more closely. Each year, CMS uses a normalization factor to scale the risk scores before payment. When CMS trains the risk adjustment model using historical data, the HCC scores are standardized such that the average risk score in traditional Medicare is 1. However, the risk profile of traditional Medicare beneficiaries changes over time, resulting in increases in the raw risk scores. A normalization factor is therefore needed to deflate the risk scores such that the traditional Medicare average risk score is 1. This is particularly important in the context of comparing V24 and V28 scores, given that they are trained on 2014-2015 data and 2018-2019 data, respectively.^3,17^ As a result, raw V24 risk scores have had more time to grow and are considerably higher. We used CMS published 2024 normalization factors for V24 (1.146) and V28 (1.015).^2^ We divide raw scores by the relevant normalization factor to arrive at the normalized risk score.

Second, we used the Demographic Estimate of Coding Intensity (DECI) as a measure of contract-level coding intensity. DECI is the measure used by MedPAC to assess coding intensity. Their past work has shown that it produces similar estimates to alternative measures, like those based on risk score changes for beneficiaries who switch from traditional Medicare to MA.^19^ It is also used by other researchers to measure coding intensity.^18,20^ DECI uses the contract's average risk score and the contract's average risk score using only demographic factors, as well as the traditional Medicare average risk score and traditional Medicare average risk score using only demographic factors. It is a ratio of ratios: the ratio of contract average risk score over traditional Medicare average risk score over the contract average demographic-only risk score over the traditional Medicare average demographic-only risk score.^18,19^ Higher DECI values indicate greater coding intensity.

Finally, we used contract characteristics to understand which types of contracts are more exposed to the new risk adjustment model, including the type of contract (eg, health maintenance organization, preferred provider organization), the contract star rating, and the share of beneficiaries who are Black, Hispanic, dually eligible for Medicaid, and eligible for Medicare because of disability.

### Analysis

Our analysis had 3 steps. First, we examined differences in V28 and V24 risk scores across insurers. We express the difference in risk scores as a percentage of V24, which is a proxy of the ultimate difference in payments across models. Second, we examined differences across contract characteristics. Finally, we examined the relationship between contract level exposure to the new risk score model (V28 score minus V24 score) as a function of the contract's DECI score. We did so using a binned scatter plot and an unadjusted ordinary least squares regression. Contracts were weighted by enrollment.

### Supplemental analysis

Insurers may change their risk coding practices over time. While full data on the implementation of V28 is not yet available, we supplement our main analysis with an examination of insurer-level trends in risk scores through 2024, the first year of the V28 phase-in. To do so, we use publicly-available plan-level payment and enrollment data to examine enrollment-weighted averages across insurers.^21,22^

### Limitations

Our study has limitations. First, while DECI has been used extensively in research and by MedPAC as a measure of coding intensity, there are fewer studies that have used DECI at the contract level where it may also be affected by differences in underlying prevalence of disease.^23^ There is no evidence however that this would render DECI unable to capture meaningful differences in coding intensity at the contract level. As a result, it has been used in past research to examine variation in coding intensity, most notably by MedPAC.^24^ Second, we lack data on risk scores after the new model was fully phased in. While we examined differences in pre-implementation scores between the new and old models, the ultimate effects of the new risk adjustment model will depend on how insurers change their risk coding practices in response to this policy change. Moreover, the changes in spending will also depend on other factors, including the participation of insurers across geographic markets, enrollment responses, and changes in bids. Further research on the effects of the V28 model will be needed as more data becomes available.

## Results

**Figure 1.**
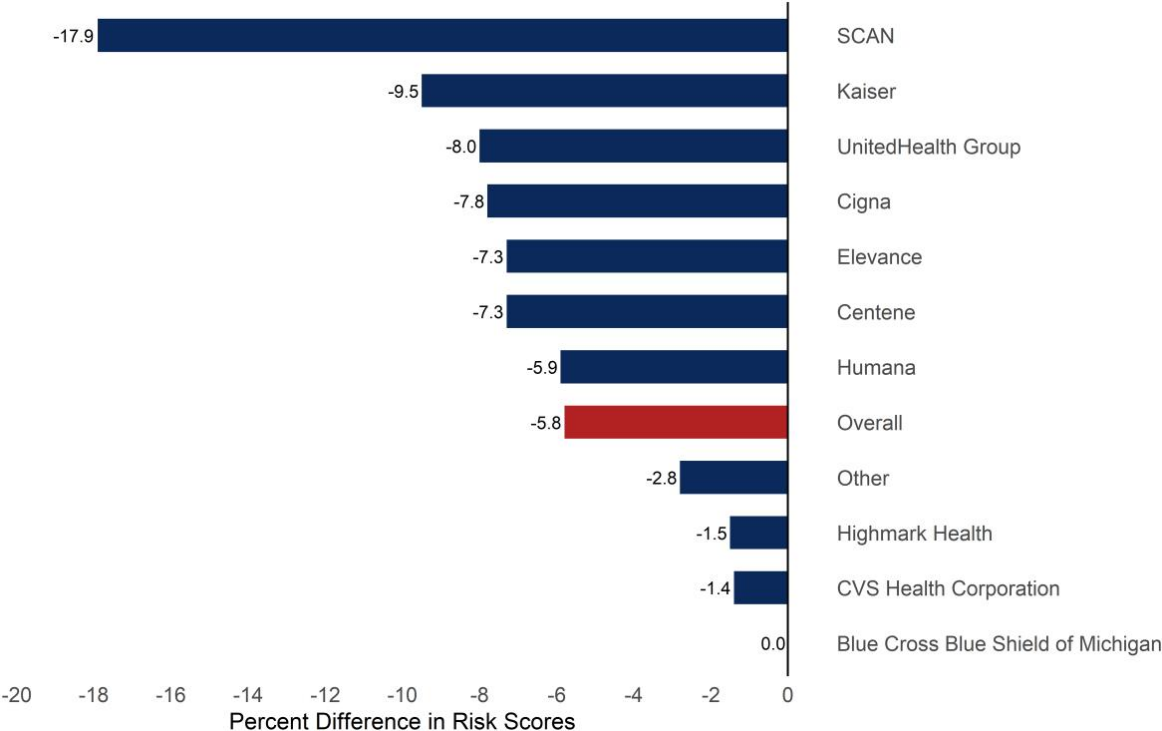
Percent differences in V28 and V24 risk scores across insurers, 2021. Source/Notes: Authors' analysis of Medicode. Values are the percent difference in risk score: 100 * (V28 − V24)/V24. Lower values indicate that the insurer is more exposed to the policy change. Before calculating differences in V24 and V28 scores, each insurer's risk scores were normalized by dividing by the 2024 V28 and V24 normalization factors (see Methods). Scores were calculated using 2021 data. Only the largest 10 insurers are shown, while all other insurers are aggregated into the “other” category.

Overall, V28 scores were 5.8% lower than V24 risk scores (Figure 1). However, there was substantial variation across insurers. For example, SCAN's V28 scores were 17.9% lower than their V24 scores. On the other hand, Blue Cross Blue Shield's V24 and V28 scores were essentially the same. Smaller insurers (outside of the largest 10 by enrollment) had 2.8% lower V28 scores, less than the overall decline. Appendix Table S1 shows insurer risk scores, the difference in scores, and the percentage difference. In supplemental analysis in Appendix Figure S1 and Appendix Table S2, we show that risk scores declined for some of the insurers with the largest exposure to V28 in Figure 1, including SCAN, Kaiser, and Cigna, while increasing for other insurers that were less exposed to V28 according to Figure 1, including Blue Cross Blue Shield of Michigan and CVS.

**Figure 2.**
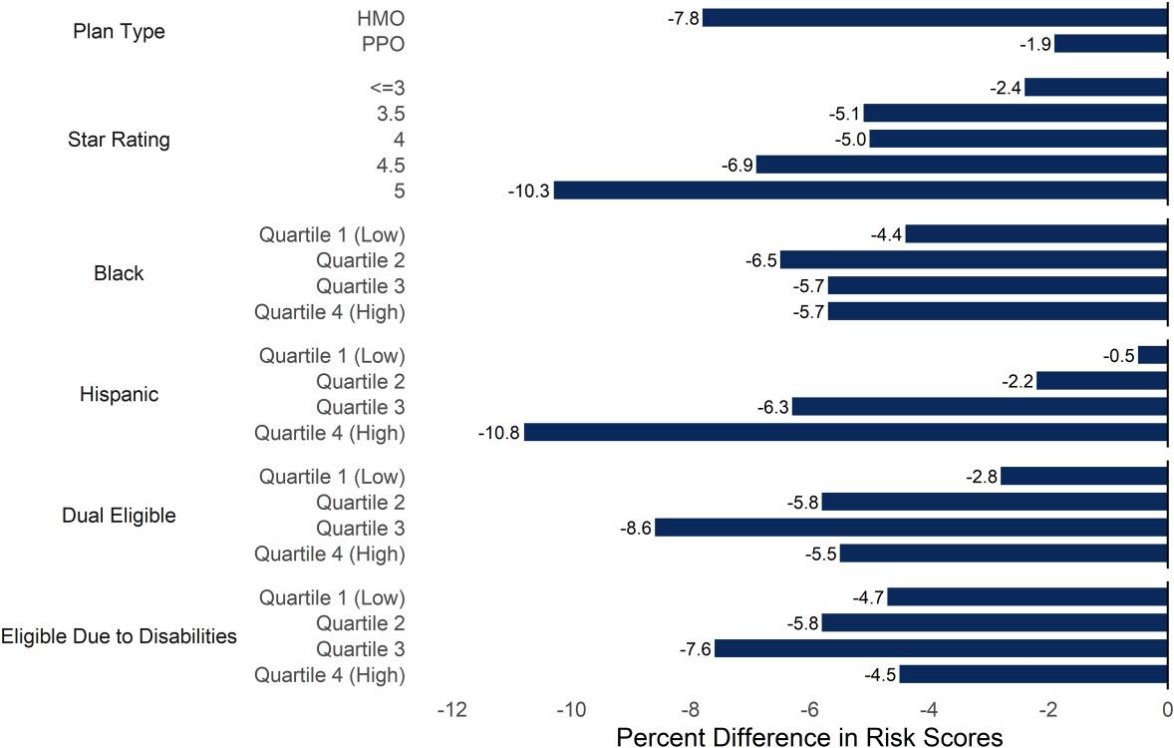
Percent differences in V28 and V24 risk scores across contract characteristics. Source/Notes: Authors' analysis of Medicode, Centers for Medicare and Medicaid Services public use data, and the Medicare Master Beneficiary Summary File. Values are the percent difference in risk score: 100 * (V28 − V24)/V24. Lower values indicate that the insurer is more exposed to the policy change. Before calculating differences in V24 and V28 scores, each contract's risk scores were normalized by dividing by the 2024 V28 and V24 normalization factors (see Methods). Scores were calculated using 2021 data. We divided contracts into quartiles based on the share of beneficiaries who were Black, Hispanic, dually eligible, and eligible due to disabilities. Values for contracts with other plan types or where 5-star ratings are not calculated by CMS are not shown.

There is also variation in the exposure to the new risk adjustment model across other contract characteristics (Figure 2). For example, HMO's had 7.8% lower V28 risk scores while PPO's had 1.9% lower V28 risk scores. 5 star rated plans had the largest percentage difference between V28 and V24 scores (10.3%). In addition, contracts in the highest quartile of enrollment by Hispanic beneficiaries had higher exposure to V28 (10.8%) than contracts with lower shares of enrollment by Hispanic beneficiaries. Appendix Table S3 shows risk scores, the difference in scores, and the percentage difference across these characteristics.

**Figure 3.**
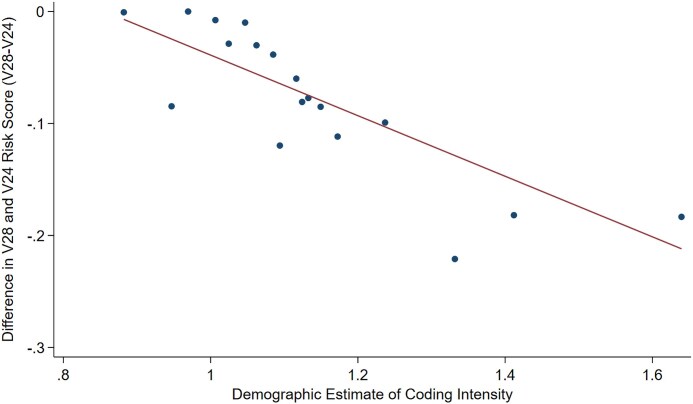
Association between estimated coding intensity and exposure to the new risk adjustment model. Source/Notes: Authors' analysis of Medicode. Values are averages of the demographic estimates of coding intensity (DECI) and the difference in V28 and V24 risk scores for evenly sized bins based on values of DECI. Before calculating differences in V24 and V28 scores, each contract's risk scores were normalized by dividing by the 2024 V28 and V24 normalization factors (see Methods). Contract-level observations were weighted by enrollment. DECI, V24 risk scores, and V28 risk scores were calculated using 2021 data.

Insurers with higher coding intensity estimates had larger differences between their V28 and V24 risk scores (Figure 3). A 0.1 percentage point higher coding intensity estimates was associated with a 0.03 percentage point larger difference between V24 and V28 scores (*P* < 0.001).

## Discussion

In this paper, we show that the new MA risk adjustment model is likely to lead to major changes in the allocations of payments across the MA program. First, using 2021 data, we find that overall risk scores are lower under the new risk adjustment system. We estimate that, without changes to coding practices, MA payments will decline by 5.8% after the full implementation of the new model. This finding is consistent with recent research by MedPAC, CMS, and other researchers.^15,24,25^ Second, exposure to the new risk adjustment model varies considerably across insurers. For example, Blue Cross Blue Shields of Michigan's risk scores were essentially unchanged when using V24 or V28. However, SCAN's risk scores were 18% lower using V28 and UnitedHealthcare's risk scores were 8% lower. Third, there were also differences in exposure based on other contract characteristics. For example, HMOs had larger differences between V24 and V28 risk scores than PPOs and 5-star rated plans had larger differences than plans with low star ratings, suggesting that payment policy changes can have distributional effects across contracts. Finally, contracts that had higher estimated coding intensity had larger differences in V28 and V24 risk scores. This finding is also consistent with recent research by MedPAC.^24^

Coding intensity is a major policy issue in the MA program. MedPAC estimates that the federal government will spend $40 billion in 2025 on payments to MA plans that are the result of greater coding intensity in MA than in traditional Medicare.^2^ The new risk adjustment model was explicitly designed to reduce the impact of coding intensity on overall spending. For example, the model modified or removed conditions that CMS believed were being coded to a greater extent in MA, including protein calorie malnutrition and diabetes with complications.^2,17^ Our results suggest that these changes are likely to reduce federal spending, though the ultimate impact depends on how insurers adapt their coding practices.

Our finding of larger differences in V28 and V24 risk scores for contracts with greater coding intensity suggests successful targeting across the MA program. A key feature of coding intensity in MA is that it varies substantially across contracts/insurers.^2,13^ This is problematic for 2 reasons. First, spending on payments to insurers with high coding intensity increases federal spending. Second, it gives disproportionate competitive advantages to some insurers that have the resources to invest in increasing coding. In recent rulemaking, CMS explicitly highlighted how this disadvantages some plans.^26^ Our results suggests that risk adjustment model revisions may help reduce payment differentials across insurers due to coding intensity.

The new risk adjustment model represents a substantial reduction in revenue for health insurers. Depending on how insurers change their business practices, this may reduce their profitability. For example, UnitedHealthcare explicitly cited the “headwinds” from the V28 model implementation as a “price reduction” and a reason for its reduced earnings. Insurers may also respond in 2 ways. First, insurers may try to adapt coding practices to the new model, as they have after past risk adjustment model revisions.^2^ However, our supplemental analysis provides some evidence that risk scores declined in the first year of the V28 phase-in for some of the insurers most exposed to the policy change, suggesting that insurers were not able to immediately adapt their coding practices. Second, insurers may change their offerings, including by increasing premiums or cost-sharing, changing networks or prior authorization practices, and/or reducing supplemental benefits. Past research on the effects of revenue shocks to MA plans has found modest reductions in plan generosity.^27,28^ Understanding the impact of this payment change on future benefit offerings will be an important area of future study as the model is fully implemented.

## Conclusion

In 2024, CMS began phasing in a new risk adjustment model to make MA payments less susceptible to higher coding intensity by MA plans. Our results suggest that this model will reduce federal spending on MA, with targeted reductions for contracts with greater initial coding intensity.

## Supplementary Material

qxag092_Supplementary_Data
