# Supplementary material for: Exposure to the new Medicare Advantage risk adjustment model varies across insurers
Source: Health Aff Sch. 2026 Apr 16;4(5):qxag092. doi: 10.1093/haschl/qxag092 (PMC13143170; doi:10.1093/haschl/qxag092)
Supplement: qxag092_Supplementary_Data [file qxag092_supplementary_data.zip › V28 Appendix Revised.docx]

**Appendix Table 1. Risk Scores by Parent**

| **Parent** | **V24** | **V28** | **Difference** | **Percent Difference** | **DECI** |
| --- | --- | --- | --- | --- | --- |
| SCAN Group | 1.431 | 1.174 | -0.257 | -17.9 | 1.178 |
| UnitedHealth Group | 1.330 | 1.224 | -0.106 | -8.0 | 1.186 |
| CIGNA | 1.342 | 1.238 | -0.104 | -7.8 | 1.196 |
| Kaiser Foundation Health Plan | 1.063 | 0.962 | -0.101 | -9.5 | 0.942 |
| Anthem Inc. | 1.383 | 1.282 | -0.101 | -7.3 | 1.231 |
| Centene Corporation | 1.326 | 1.230 | -0.096 | -7.3 | 1.191 |
| Humana Inc. | 1.244 | 1.170 | -0.074 | -5.9 | 1.123 |
| Overall | 1.240 | 1.169 | -0.072 | -5.8 | 1.122 |
| Other | 1.179 | 1.146 | -0.033 | -2.8 | 1.087 |
| Highmark Health | 1.170 | 1.152 | -0.018 | -1.5 | 1.070 |
| CVS Health | 1.131 | 1.115 | -0.016 | -1.4 | 1.055 |
| Blue Cross Blue Shield of Michigan | 1.112 | 1.112 | 0.000 | 0.0 | 1.043 |

SOURCE Authors’ analysis of Medicode. NOTES Each insurer’s risk scores were normalized by dividing by the 2024 V28 and V24 normalization factors (see Methods). Scores were calculated using 2021 data. Only the largest 10 insurers are shown, while all other insurers are aggregated into the “other” category. Differences may not sum together due to rounding.

**Appendix Table 2. Trends in Risk Score by Insurers, 2023-2024**

| **Insurer** | **Risk Score** | | **Percent Change** |
| --- | --- | --- | --- |
|  | **2023** | **2024** |  |
| Cigna | 1.161 | 1.116 | -3.9 |
| SCAN | 1.272 | 1.225 | -3.7 |
| Kaiser | 0.964 | 0.945 | -1.9 |
| Elevance (formerly Anthem) | 1.270 | 1.268 | -0.2 |
| Humana | 1.143 | 1.145 | 0.2 |
| Other | 1.079 | 1.087 | 0.7 |
| Overall | 1.140 | 1.150 | 0.9 |
| Highmark | 1.068 | 1.081 | 1.2 |
| United | 1.219 | 1.235 | 1.4 |
| Blue Cross Blue Shield of Michigan | 1.032 | 1.061 | 2.8 |
| Centene | 1.203 | 1.249 | 3.9 |
| CVS | 1.036 | 1.086 | 4.8 |

SOURCE Authors’ analysis of publicly-available centers for Medicare and Medicaid Services plan payment and enrollment data. NOTES 2024 was the first year of the partial phase-in of the V28 risk adjustment model. Values are enrollment-weighted average Hierarchal Condition Category scores for each insurer.

**Appendix Table 3. Risk Scores by Contract Characteristics**

| **Contract Characteristics** | **V24** | **V28** | **Diff.** | **Stat.**  **Sig** | **Percent Diff.** | **DECI** |
| --- | --- | --- | --- | --- | --- | --- |
| Plan Type |  |  |  |  |  |  |
| HMO | 1.302 | 1.201 | -0.102 | Ref. | -7.8 | 1.160 |
| PPO | 1.133 | 1.113 | -0.021 | *** | -1.9 | 1.055 |
| MSA | 0.612 | 0.657 | 0.044 | *** | 7.2 | 0.647 |
| PFFS | 1.226 | 1.227 | 0.001 | *** | 0.1 | 1.075 |
| Star Rating |  |  |  |  |  |  |
| <=3 | 1.218 | 1.188 | -0.029 | Ref. | -2.4 | 1.123 |
| 3.5 | 1.265 | 1.201 | -0.064 | * | -5.1 | 1.150 |
| 4 | 1.232 | 1.169 | -0.062 | * | -5.0 | 1.118 |
| 4.5 | 1.263 | 1.176 | -0.087 | * | -6.9 | 1.130 |
| 5 | 1.193 | 1.070 | -0.123 | ** | -10.3 | 1.047 |
| Not Calculated | 1.246 | 1.193 | -0.054 | * | -4.3 | 1.157 |
| Black |  |  |  |  |  |  |
| Quartile 1 (Low) | 1.18 | 1.128 | -0.052 | Ref. | -4.4 | 1.076 |
| Quartile 2 | 1.200 | 1.122 | -0.078 |  | -6.5 | 1.077 |
| Quartile 3 | 1.27 | 1.198 | -0.072 |  | -5.7 | 1.151 |
| Quartile 4 (High) | 1.345 | 1.268 | -0.077 |  | -5.7 | 1.22 |
| Hispanic |  |  |  |  |  |  |
| Quartile 1 (Low) | 1.104 | 1.098 | -0.006 | Ref. | -0.5 | 1.033 |
| Quartile 2 | 1.154 | 1.129 | -0.025 | * | -2.2 | 1.072 |
| Quartile 3 | 1.262 | 1.183 | -0.079 | ** | -6.3 | 1.137 |
| Quartile 4 (High) | 1.384 | 1.234 | -0.15 | *** | -10.8 | 1.206 |
| Dual |  |  |  |  |  |  |
| Quartile 1 (Low) | 1.154 | 1.122 | -0.032 | Ref. | -2.8 | 1.063 |
| Quartile 2 | 1.136 | 1.07 | -0.066 |  | -5.8 | 1.031 |
| Quartile 3 | 1.339 | 1.224 | -0.115 | ** | -8.6 | 1.191 |
| Quartile 4 (High) | 1.620 | 1.531 | -0.089 | ** | -5.5 | 1.450 |
| Eligible Due to Disabilities |  |  |  |  |  |  |
| Quartile 1 (Low) | 1.148 | 1.094 | -0.054 | Ref. | -4.7 | 1.042 |
| Quartile 2 | 1.199 | 1.131 | -0.069 |  | -5.8 | 1.093 |
| Quartile 3 | 1.337 | 1.235 | -0.102 | * | -7.6 | 1.194 |
| Quartile 4 (High) | 1.469 | 1.403 | -0.066 |  | -4.5 | 1.335 |

SOURCE Authors’ analysis of Medicode, Centers for Medicare and Medicaid Services public use data, and the Medicare Master Beneficiary Summary File. NOTES Each contract’s risk scores were normalized by dividing by the 2024 V28 and V24 normalization factors (see Methods). Scores were calculated using 2021 data. We divided contracts into quartiles based on the share of beneficiaries who were Black, Hispanic, dually eligible, and eligible due to disabilities. Differences may not sum together due to rounding. P-values were obtained using enrollment-weighted contract-level bivariate linear regressions of V28 exposure(V28 risk score-V24 risk score)on the given categorical variable. * p < 0.05, ** p < 0.01, *** p < 0.001

**Appendix Figure 1. Trends in Risk Score Among Selected Insurers, 2021-2024**


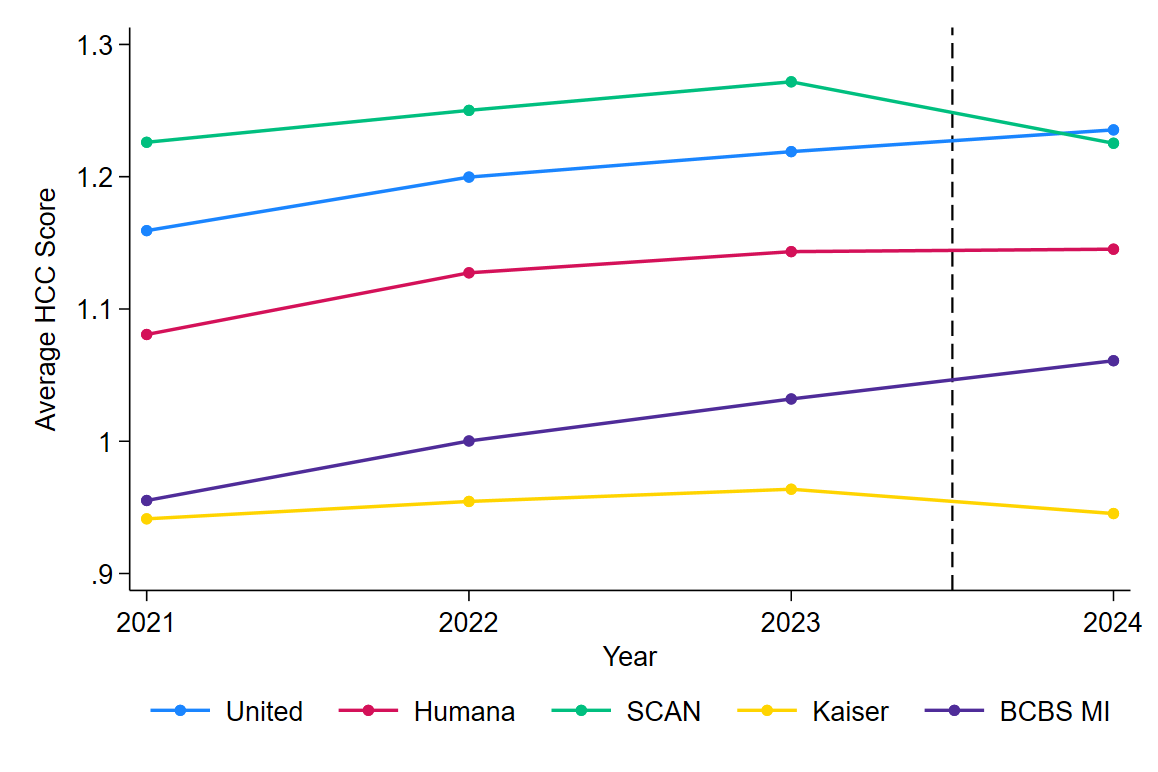


SOURCE Authors’ analysis of publicly-available centers for Medicare and Medicaid Services plan payment and enrollment data. NOTES The vertical line indicates the first year of the partial phase-in of the V28 risk adjustment model. Values are enrollment-weighted average Hierarchal Condition Category scores for each insurer. Abbreviations: HCC, Hierarchal Condition Category; BCBS MI, Blue Cross Blue Shield of Michigan.
